# A fish-parasite sentinel system in an assessment of the spatial distribution of polychlorinated biphenyls

**DOI:** 10.1038/s41598-023-31939-4

**Published:** 2023-03-30

**Authors:** Mikuláš Oros, Daniel Barčák, Dana Miklisová, Dalibor Uhrovič, Tímea Brázová

**Affiliations:** Institute of Parasitology, Slovak Academy of Sciences, Košice, Slovakia

**Keywords:** Zoology, Environmental sciences

## Abstract

The spatial distribution of polychlorinated biphenyls (PCBs), in the Zemplínska Šírava water reservoir and adjacent tributaries in the Bodrog River Basin were investigated using a fish-parasite sentinel system. PCB concentrations were detected in various fish matrices (dorsal and abdominal muscles, liver and intestine) of the Wels catfish (*Silurus glanis*) and its intestinal cestode *Glanitaenia osculata.* PCB concentrations in the fish from the water reservoir, located closest to the chemical plant, the primary source of the PCB pollution, were the highest. The analysis of these contaminants in catfish matrices showed the highest concentrations in the abdominal muscle, followed by the dorsal muscle, liver and intestine. Concentrations of ∑PCBs exceeding the limits for food set by European regulations were measured in the muscle tissue of catfish at all sites, even in the Bodrog River, 60 km away from the primary source of contamination, posing a significant risk to humans in the Zemplín region. For the first time, the ability of cestode *G. osculata* to accumulate higher amounts of PCBs compared to fish matrices has been demonstrated. Due to the enormous ability of the parasites to accumulate PCBs, we recommend this approach for alternative biomonitoring of PCBs in contaminated aquatic environments.

## Introduction

Persistent organic pollutants (POPs), including polychlorinated biphenyls (PCBs), are ubiquitous anthropogenic contaminants that are resistant to degradation in the environment. Due to their lipophilic nature, they accumulate in adipose tissue and increase in concentration as they move up the food chain^1^, thus triggering multiple adverse effects in organisms^2^. Although the production of PCBs was banned many years ago, they can still enter the environment today through poorly maintained, mostly illegal hazardous waste sites or leaks from various electrical appliances containing PCBs^3^ and during the unintentional generation of PCBs as by-products of pigment and dye production^4,5^. The group of PCBs consists of a total of 209 individual chlorinated components (so-called congeners)^6^. However, Commission Regulation (EC)^7^ recommends the use of six PCBs (PCB 28, PCB 52, PCB 101, PCB 138, PCB 153 and PCB 180) as indicator PCBs in monitoring studies. Their sum is considered to be an appropriate marker for occurrence and human exposure to PCBs and should therefore be set as a maximum level.

The Zemplínska Šírava water reservoir is one of the largest artificial water reservoirs in Slovakia. However, in the past, a large amount of waste containing PCBs was released from a former chemical factory in the nearby town of Strážske into the Laborec River, which is the main tributary source of this reservoir. This resulted in the severe contamination of sediments, surrounding soils, surface and underground waters and food chains in the region^8^. Another and still active sources of contamination are barrels with PCBs produced under the brand names Delor, Delotherm and Hydelor, which were buried near the factory after their production was banned. As a consequence, the Slovak government declared a state of emergency in this region in early 2020.

The latest strict regulations only allow the “catch and release” of fish from the reservoir; their consumption is prohibited. The high level of pollution in the reservoir also means that the biota in the Laborec River and in the nearby Latorica Protected Landscape Area belonging to the Bodrog River Basin is also very likely to be negatively affected. Information about the ecological state of a protected landscape area is always extremely important. Therefore, it is surprising that no integrated data on the spatial transfer of hazardous substances from the Zemplínska Šírava reservoir to the area of the Bodrog watershed are available so far.

Fish are often used as bioindicators to monitor environmental pollution because they are close to the top of the food chain in aquatic environment and have a relatively long life span^9^, which allows them to store high amounts of contaminants. In our study, we used the Wels catfish (*Silurus glanis*) for this purpose. Its benthic way of life and broad-spectrum diet as well as its high position in the food chain and potential to accumulate high concentrations of organic pollutants compared to the rest of the aquatic community make this fish a good candidate for pollution monitoring^10^. *S. glanis* is an economically important species in commercial, recreational and aquaculture fisheries^11^. In Slovakia, the Wels catfish inhabits mainly fish-rich, weedy lakes and water reservoirs as well as slow, deep lowland rivers (e.g. the Danube, Tisa and Bodrog River Basins)^12^. This species is a frequent and abundant fish in Slovak rivers and reservoirs.

Numerous studies have demonstrated that cestodes have a higher accumulation potential than their hosts [e.g.^13–16^]. All of these studies, however, mainly identified these parasites as useful sentinels of heavy metals, while studies on the bioaccumulation of PCBs, in fish cestodes are still very limited^17–19^. The cestode *Glanitaenia osculata* (Goeze, 1782) is a specific parasite of the Wels catfish reported from several countries in Europe and Palaearctic Asia^20^. Our group of catfish was also examined parasitologically, and this tapeworm was found quite frequently in their intestines. Subsequently, we used this fish-parasite model to assess the concentration and spatial distribution of PCBs at the sites studied.

The aim of the present study is to determine the concentrations of the six indicator congeners in Wels catfish and its specific parasite in order to assess the reliability of this parasite–host model as a suitable bioindicator of organic pollution. In particular, the accumulation of PCBs in various fish matrices, their distribution in different water bodies, and the influence of age, tissue lipid content and parasitic infection are discussed.

## Results

### Polychlorinated biphenyls in Wels catfish matrices

The lipid content (mean % ± SD) of the catfish matrices and parasites were as follows: dorsal muscle 0.75 ± 1.15 (0.03–6.2%), abdominal muscle 1.2 ± 1.83 (0.05–8.9%), liver 0.48 ± 0.52 (0.03–2.25%), intestine 0.89 ± 1.05 (0.05–4.9%) and cestodes 1.57 ± 1.12 (0.13–3.76%).

The tissue analysis of six PCB congeners in four catfish matrices showed the highest concentrations in abdominal muscle (∑PCB 154 ± 318 ng g^−1^ wet weight—w.w.), followed by dorsal muscle, liver and intestine (Table 1). The concentrations found in both muscle types were comparatively high (Wilcoxon paired test: Z = 0.36, p ˃ 0.05, N = 27) and were not significantly different than those in the liver (p ˃ 0.05).

**Table 1:** Mean concentrations of individual PCB congeners and their sum (mean ± SD) in fish and their parasites (ng g^−1^ w.w.) from four examined localities.

	Muscle D	Muscle A	Liver	Intestine	*G. osculata*
	N = 47	N = 30	N = 47	N = 47	N = 12
PCB 28	1.38 ± 2.96	3.59 ± 8.42	1.18 ± 3.14	1.09 ± 1.71	6.17 ± 8.73
PCB 52	3.90 ± 9.49	10.4 ± 24.1	3.06 ± 8.63	3.44 ± 4.11	22.0 ± 35.7
PCB 101	5.88 ± 15.3	14.2 ± 31.1	6.69 ± 19.2	10.5 ± 16.9	63.0 ± 92.2
PCB 138	20.6 ± 60.7	43.1 ± 88.1	15.2 ± 42.6	13.1 ± 14.3	184 ± 281
PCB 153	23.9 ± 69.9	51.0 ± 105	19.6 ± 55.0	14.6 ± 16.2	224 ± 328
PCB 180	15.4 ± 47.7	31.3 ± 64.3	11.2 ± 34.3	7.98 ± 9.18	131 ± 192
Ʃ PCB	71.0 ± 204	154 ± 318	57.0 ± 161	50.7 ± 54.0	631 ± 934

**Figure 1:**
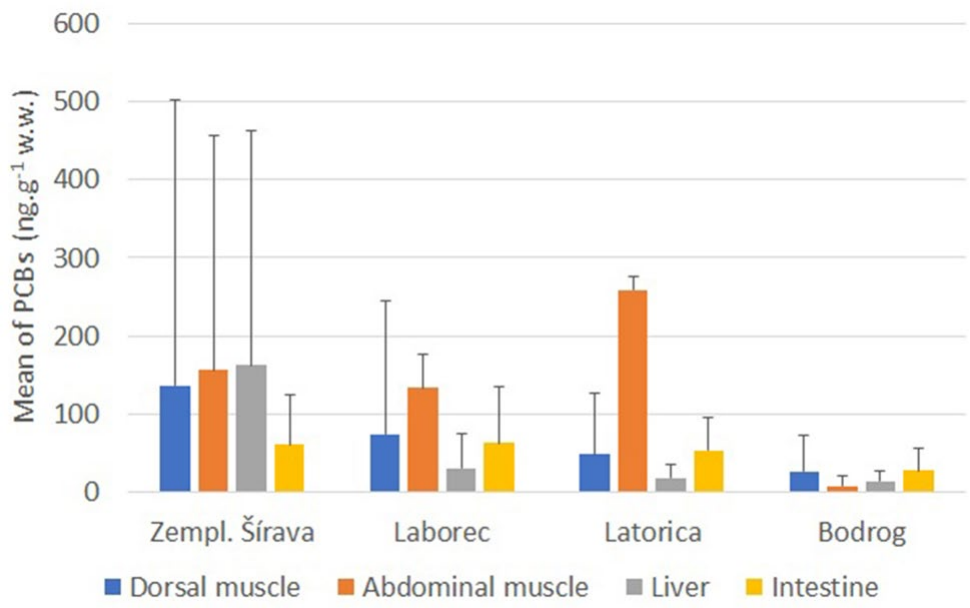
Mean concentrations of PCBs (mean ± SD) in fish matrices (ng g^−1^ w.w.) from four sampling sites.

The highest PCB concentrations occurred in the abdominal muscle of fish from the Latorica River (259 ± 550 ng g^−1^ w.w.). High PCB levels were also detected in the liver and dorsal muscle of fish from the Zemplínska Šírava reservoir (163 ± 299 and 136 ± 365 ng g^−1^ w.w., respectively). In the Laborec River downstream of the town of Strážske and the Z. Šírava reservoir, the highest amounts of PCBs was measured in the abdominal muscle (133 ± 244 ng g^−1^ w.w.). PCBs were detected even in young fish (only 1–3 years old specimens were caught) from the most distant course of the Bodrog River, but their values were considerably lower (27.2 ± 45.1 in dorsal muscle ng g^−1^ w.w.) (Fig. 1).

According to the Commission Regulation (EC)^7^ the maximum value of ∑PCB in fish meat (125 ng g^−1^ w.w.) was exceeded in 21% of cases from the individual locations, namely in 25% of fish muscle from the Zemplínska Šírava reservoir (up to 1285 ng g^−1^ w.w.), 27% of samples from the rivers Laborec (797 ng g^−1^ w.w.) and Latorica (the highest PCB concentration was 1377 ng g^−1^ w.w.) and in 8% of samples from the Bodrog River (127.5 ng.g^−1^ w.w.).

**Figure 2:**
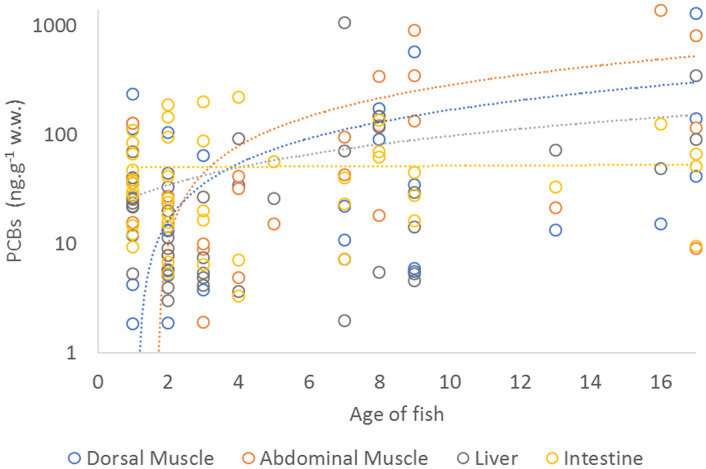
Mean concentrations of PCBs in fish matrices (ng g^−1^ w.w.) in relation to fish age.

**Figure 3:**
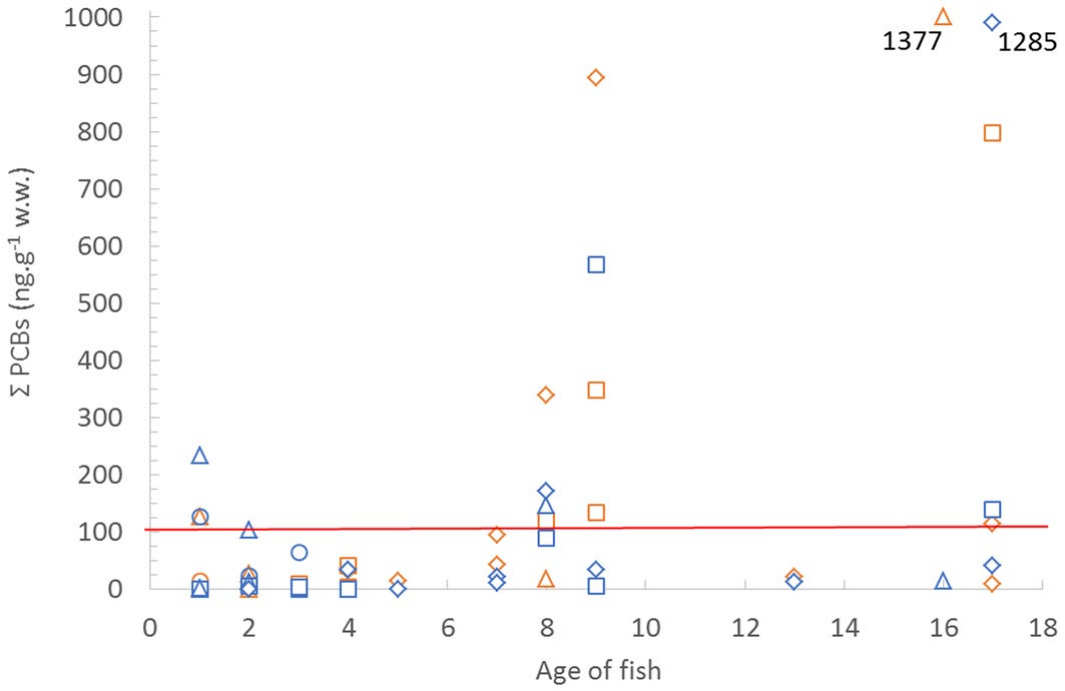
Sum of PCBs detected in dorsal and abdominal muscle in ng g^−1^ w.w in relation to age of fish (N = 30). Plus point (+) for age 16, resp. 17 represents value 1377, resp. 1285. Red line represents maximum permissible value of sum of PCB in fish meat in wet weigt (limit is ng g^−1^ w.w.).

PCB concentrations increased with the age of the fish. This trend was most evident in the dorsal (p ˂ 0.01) and abdominal muscles of the fish (p ˂ 0.001) (Fig. 2). The maximum permissible limits of PCBs in catfish prevailed from 8- to 17-year-old individuals (Fig. 3), with the highest concentrations in the oldest fish (17 years; 1377 ng g^−1^ w.w. from the Latorica River).

The impact of the following factors on PCB concentrations among individual fish was tested using GLMMs (Table 2): *lipid content* in the biological samples, *fish matrices* (dorsal muscle, liver, and intestine), *localities*, *age* and *parasitic infection*. The Mann–Whitney U-test (Z = 0.05, p = 0.96) confirmed no differences in PCB absorption between male and female fish; therefore, sex was not included in the modelling. The *age* and *lipid content* in the biological samples had a meaningful positive effect on PCB concentrations in the catfish in all four models. The effect size of the factor *lipid content* was more extensive than the factor *age*. In contrast, we detected no effect of the parasitic infection on PCB accumulation in fish, which we expected. The stand-alone levels of factors, fish matrices and locality had meaningful effects on the model outcomes. However, the uncertain intervals of conditional effects of these covariates (Supplementary Fig. S1 online) overlapped, so we could not rule out the possibility that there were, in fact, no differences among levels of each covariate.

**Table 2:** Generalized linear mixed models analysing the effects of individual traits (age, lipid content, matrices, parasite infection, locality) on the PCB concentrations. The covariates levels with credible intervals that do not overlap with zero and, therefore, can be considered meaningful predictors are highlighted in bold and italics.

Model	Covariate	Estimate	Estimate error	95% credible interval
∑PCB	Intercept	1.06	0.42	0.22–1.89
	Matrix			
	Intestine	(Ref.)	(Ref.)	(Ref.)
	***Dorsal muscle***	− 0.79	0.23	− 1.24 to − 0.34
	***Liver***	− 0.12	0.23	− 0.57 to 0.33
	***Age***	0.08	0.03	0.02–0.15
	Parasites			
	Uninfected	(Ref.)	(Ref.)	(Ref.)
	Infected	− 0.40	0.33	− 1.06 to 0.26
	Locality			
	Bodrog	(Ref.)	(Ref.)	(Ref.)
	***Zemp. Šírava***	1.08	0.43	0.24–1.93
	Laborec	− 0.42	0.45	− 1.33 to 0.47
	Latorica	0.19	0.39	− 0.58 to 0.96
	***Lipid***	0.82	0.11	0.61–1.04
PCB138	Intercept	1.18	0.40	0.38–1.96
	Matrix			
	Intestine	(Ref.)	(Ref.)	(Ref.)
	***Dorsal muscle***	− 0.77	0.23	− 1.22 to − 0.32
	Liver	0.04	0.23	− 0.41 to 0.48
	***Age***	0.08	0.03	0.02–0.14
	Parasites			
	Uninfected	(Ref.)	(Ref.)	(Ref.)
	Infected	− 0.32	0.31	− 0.94 to 0.3
	Locality			
	Bodrog	(Ref.)	(Ref.)	(Ref.)
	***Zemp. Šírava***	1.01	0.40	0.22–1.81
	Laborec	− 0.56	0.43	− 1.41 to 0.29
	Latorica	0.12	0.37	− 0.62 to 0.85
	***Lipid***	0.84	0.11	0.62–1.06
PCB153	Intercept	0.44	0.45	− 0.45 to 1.31
	Matrix			
	Intestine	(Ref.)	(Ref.)	(Ref.)
	***Dorsal muscle***	− 0.66	0.26	− 1.17 to − 0.17
	Liver	− 0.27	0.25	− 0.77 to 0.22
	***Age***	0.09	0.03	0.03–0.16
	Parasites			
	Uninfected	(Ref.)	(Ref.)	(Ref.)
	Infected	− 0.41	0.35	− 1.1 to 0.26
	Locality			
	Bodrog	(Ref.)	(Ref.)	(Ref.)
	***Zemp. Šírava***	1.34	0.45	0.47–2.23
	Laborec	− 0.50	0.48	− 1.45 to 0.45
	Latorica	0.26	0.41	− 0.55 to 1.07
	***Lipid***	0.86	0.12	0.62–1.11
PCB180	Intercept	2.14	0.47	1.22–3.05
	Matrix			
	Intestine	(Ref.)	(Ref.)	(Ref.)
	***Dorsal muscle***	− 1.05	0.26	− 1.56 to − 0.53
	Liver	− 0.19	0.26	− 0.71 to 0.33
	***Age***	0.09	0.04	0.02–0.16
	Parasites			
	Uninfected	(Ref.)	(Ref.)	(Ref.)
	Infected	− 0.40	0.36	− 1.12 to 0.31
	Locality			
	Bodrog	(Ref.)	(Ref.)	(Ref.)
	***Zemp. Šírava***	1.14	0.46	0.23–2.05
	Laborec	− 0.50	0.49	− 1.47 to 0.47
	Latorica	0.22	0.43	− 0.62 to 1.06
	***Lipid***	0.95	0.13	0.7–1.21

### Polychlorinated biphenyls in the cestode *Glanitaenia osculata*

The overall prevalence of the cestode *Glanitaenia osculata* in Wels catfish was relatively high, reaching 63.8% (30/47). The proportion of infected fish at the individual sampling sites was 83% in the Zemplínska Šírava reservoir, 18% in the Laborec, 64% in the Latorica and 85% in the Bodrog River. The mean intensity of infection ranged from 1.5 ± 0.65 (1–2) in the Laborec River to 82 ± 82.4 (4–300) in the Zemplínska Šírava reservoir (Supplementary Table S1 online). The mean concentration of PCBs (mean ± SD) in parasites at the individual sampling sites was as follows: Zemplínska Šírava reservoir 833 ± 1007 ng g^−1^ wet weight; Latorica River 32.6 ± 19.2 ng g^−1^ w.w. and Bodrog River 31.6 ± 1.4 ng g^−1^ w.w. Due to the low amount of parasites in the intestines of the Wels catfish from the Laborec River, no analysis for PCBs was performed.

**Figure 4:**
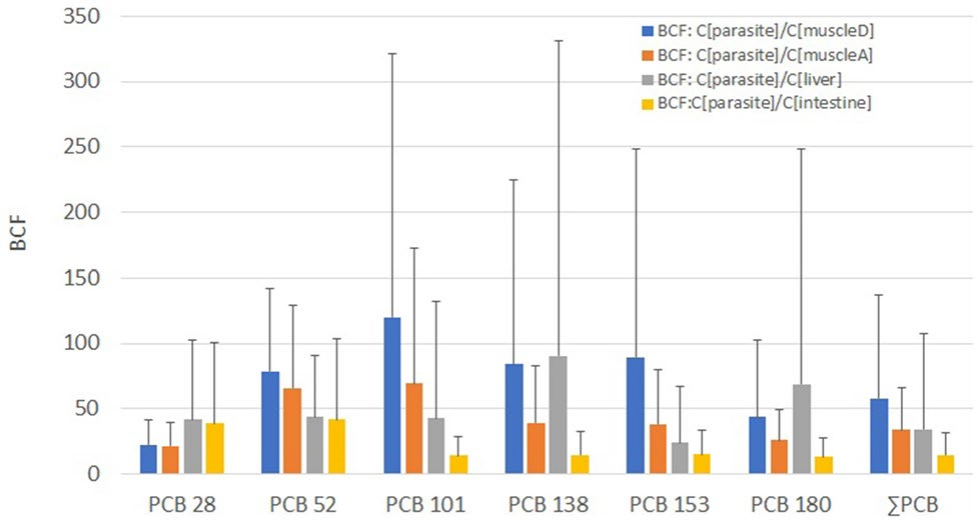
Ratios of PCB concentrations (bioconcentration factors—BCFs) (mean ± SD) (ng g^−1^ of w.w.) in *G. osculata* relative to that in fish matrices. *MuscleD*—dorsal muscle, *MuscleA*—abdominal muscle.

This study pointed out that *G. osculata* is able to concentrate much higher amounts of PCBs compared to the studied fish matrices (Wilcoxon pairs test, Fig. 4), which also corresponded to the bioconcentration factors (BCF), whose calculated value was always above 1. The accumulation potential of the tapeworm was best demonstrated against the dorsal muscle, where the BCF with congener PCB 101 reached a value of 120. Conversely, the lowest BCF was recorded for the tapeworm and fish intestine (BCF = 15, Fig. 4). Despite the high ability of *G. osculata* cestodes to absorb and accumulate PCBs in their tissues, we detected no effect of the parasitic infection on PCB accumulation in fish (Table 2).

We also observed that female catfish harboured greater numbers of *G. osculata* tapeworms (mean ± SD; 88 ± 113) than males (mean ± SD; 14 ± 21) (Supplementary Table S1 online), but sex differences in the intensity of infection did not significantly affect the PCB accumulation in the tissues of fish hosts. Neither fish size nor age had an effect on the infection of fish with this parasite (Spearman’s correlation analysis, p ˃ 0.05). On the contrary, the intensity of infection significantly increased with the increasing PCB concentrations in fish intestine (Spearman’s correlation analysis, p ˂ 0.05).

## Discussion

Aquatic sediment is the main sink for pollutants, including PCBs, whose concentration can reach significant levels over time due to their resistance to natural degradation. They can enter plants and animals at the bottom of the food chain, then be consumed by animals, moving up the same chain. The chemicals found in animals and plants then enter the human body through food^21^. During regular monitoring of the Zemplínska Šírava reservoir from 1999 to 2007, high levels of PCB indicator congeners (up to 1633 μg kg^−1^ dry weight) were measured in bottom sediments^22^. It is assumed that at least 40,000 tons of PCB-contaminated sediments are still present in the reservoir and in the sediments in the waste water canals^23,24^. Even though no recent complex measurements of PCBs in the sediments have been made, we do not assume a significant reduction of the contamination by natural attenuation, as no remediation measures have been implemented yet. Barrels filled with PCBs, which were buried near the factory after their production was banned, are still active source of contamination.

Moreover, several studies over the last 35 years have shown that elevated concentrations of PCBs in the Zemplínska Šírava reservoir pose a potential risk to aquatic health, especially to fish and bottom-dwelling invertebrates, which are the main food source for fish^25–27^. Negative impacts on the resident population in the Zemplín region have also been repeatedly documented^28–30^. For example, the Zemplín region has a significantly higher incidence of various cancers, endocrine disorders and developmental defects in foetuses compared to the average Slovak population, all of which appear to be associated with increased concentrations of OH-PCBs in the blood serum of adults and children from this area^31^.

The excessive PCB concentrations determined in the meat of Wels catfish in the present study, and previously in other fish species from the Zemplínska Šírava reservoir^19,25,32^, confirm that pollution by PCBs is still serious. It is worth noting that the area around the reservoir is a well-known centre for various outdoor activities, especially swimming, fishing and eating fish. Our findings are therefore of crucial importance, especially with regard to human health and the quality of life in the Zemplín region.

Aside from the Zemplínska Šírava reservoir, surprisingly high concentrations of PCBs were also measured in the abdominal muscle of catfish from the Latorica River, with a value about 11-times higher than the maximum limit stated for human consumption. Our findings are important because this locality is designated as an internationally important wetland (see^33^). From the present results, it is apparent that this territory, consisting of a system of arms surrounded by floodplain forest with a unique water and swamp biocenosis and previously considered “untouched” by pollution, is particularly threatened by PCB contamination.

The lowest PCB values were measured in fish matrices from the Bodrog River, which could be related to its greatest distance from the pollution source, but also to the fact that only young, 1–3-year-old fish from this location were analysed.

Previous studies^19,34,35^ have pointed out that the amounts of PCBs accumulated in fish are dependent on various factors, such as lipid content, seasonality or the age of the fish. As a fish’s age increases, its lipid content also increases^36^. Since polychlorinated biphenyls are lipophilic substances, they tend to accumulate in lipid rich matrices. This is consistent with the results of our analyses, as the highest concentrations of PCBs were found in the abdominal muscle, i.e. the tissue with the highest lipid content in the oldest catfish (17 years old), with maximum concentrations of 1377 ng g^−1^ w.w. Mikolajczyk et al.^37^ studied PCB accumulation in predatory and non-predatory fish species. They found that pike, zander and catfish, which were the leanest fish, contained low levels of PCBs in contrast to fatty fish such as bream or roach. Our results of PCB contamination in the flesh of Wels catfish from the present study and previously from freshwater bream^19^ are consistent with these findings. Although the PCB concentrations in bream muscle were lower (39 ng g^−1^ w.w.) than PCB levels in catfish (163 ng g^−1^ w.w.), the adipose tissue of the bream contained 20,600 ng g^−1^ lipid weight.

Although previous studies evidenced a seasonal effect of PCB accumulation in fish tissues [e.g.^35^], this phenomenon, however, could not be assessed in our study, because the fish were collected only in the spring and early summer (May and June), when the biohelminths (i.e. with complex life cycle) used for the bioaccumulation study are most abundant. Nevertheless, no differences were found in terms of PCB content in the fish matrix and parasites in the 2 years studied.

Although the PCB concentrations in fish from the most distant sampling site (Bodrog River) were relatively low compared to three other localities, they nevertheless exceeded the limit concentrations stated for human consumption in fish muscle. The possible reason for the high concentrations in fish caught even 60 km away from the original source of pollution (the Strážske chemical plant) may be the territorial behaviour and downstream migration of *S. glanis*, as previously observed by Kuzishchin et al.^38^ in the lower Volga River system. Although the Wels catfish is not a typical migratory species, it cannot be ruled out that fish with the heaviest PCB loads in their tissues escaped from the Zemplínska Šírava reservoir through the drainage channel into the Laborec River and further into the rivers of the Bodrog River Basin. Moreover, older fish may have pushed younger fish out of the reservoir. Thus, it is possible that fish with high levels of PCBs from the lower parts of the Bodrog Basin originate from the reservoir, after crossing migration barriers and settled further downstream. However, this fact does not mitigate the health risk associated with the consumption of fish, especially older fish, and calls for similar restrictions on the consumption of fish meat as those introduced in the Zemplínska Šírava reservoir (“catch and release”).

Studies addressing the storage of organic compounds in fish cestodes are understudied^17–19^ compared to the large number of data on the accumulation of various metals in the fish-tapeworm (host–parasite) system^14–16,32^. In the present study, we investigated and compared the accumulation of PCB compounds in the tapeworm *G. osculata* and its specific host, the Wels catfish. The tapeworm *G. osculata*, like other tapeworm species, absorbs nutrients from the intestine of the fish host over its entire body surface. Therefore, it is likely that it also absorbs harmful pollutants, inorganic and organic compounds in the same way, namely by diffusion, mediated diffusion and active transport from the host^39^. As expected, the cestodes accumulated significantly, up to 30–60 times higher amounts of these contaminants compared to the fish matrices, with the difference being greatest in fish muscle. These results are important, because fish muscle is the main material used for aquatic pollution assessment^40^. However, the concentrations of PCBs or other pollutants in fish muscle may not accurately reflect the levels of these pollutants in the environment, as demonstrated in this or our previous studies, where parasites accumulated higher amounts of PCBs compared to their fish hosts^19,32^. Therefore, we recommend these parasitic species as useful bioindicator of PCBs in aquatic environments.

We have found that the rule that applies to fish, according to which PCB uptake correlates with the lipid content in the fish body or fish matrices, does not work for parasites. Despite the fact that the lipid content in the parasites was higher than in the fish matrices (1.57 ± 1.12%), the PCB concentrations measured in the parasite tissues did not correlate with the lipid content (p ˃ 0.05). Therefore, we hypothesise that PCBs in tapeworms are taken up and transported by different pathways than in vertebrates, and these pathways are not yet known.

The interactions between parasites and their hosts in a contaminated environment are very complex and poorly understood. Parasites, together with contaminants, can cause environmental stress for the host, e.g. increasing PCB concentrations may supress the immune system of the fish, leading to an increase in parasite densities^41,42^. In the present study the parasite intensity increased with the PCB amounts in fish intestine; therefore, we hypothesise that this increase of contaminants could be caused by the immunosuppression of the catfish as was demonstrated in previous studies^41,43^.

Several literature sources^44,45^ confirm that the distribution and abundance of parasites in vertebrates are influenced by the sex of the host. In the present study, no significant differences in parasite abundance were found, although female catfish harboured a greater number of *G. osculata* cestodes than males. Mille et al.^46^ showed that larger male hake harboured a greater number of endoparasites than smaller females. They explained that this difference could be caused by sexual dimorphism in fish size. Poulin^44^ also explained that host size correlates with parasite burdens, i.e. the larger individuals exhibit higher parasite burden, irrelevant of fish sex. Our female fish were on average 9 years old and the males 6 years old and the weight of females was two times higher than that of the males (the average weight of the female fish was 20,438 g and that of the males 8764 g), confirming the assumptions of previous authors.

It has been shown that some intestinal parasites can hypothetically have a positive impact on their hosts in a contaminated environment (a “detoxification effect”) by eliminating pollutants from the tissues of infected hosts^14^. In the present study the tapeworms were able to accumulate significantly higher amounts of PCBs compared to their fish host. Although their intensity of infection was relatively high (average intensity of infection from all localities was 35 (1–300) (see Supplementary Table S1 online), a detoxification effect of *G. osculata* on its fish host could not be demonstrated, as was the case in several previous studies dealing with heavy metals (e.g.^13,14,42^), as well as PCB compounds^32^. These results highlight the importance of future experimental studies on the accumulation and observation of detoxification pathways of PCBs in this group of parasites.

As far as we know, this is the first comparative study to investigate the relationship between Wels catfish and the tapeworm *G. osculata* with respect to the accumulation and distribution of PCBs in the environment. The PCB concentrations in fish, which exceeded the toxicological threshold values of PCBs in fish meat stated for human consumption, indicate that there is still a high risk for aquatic organisms and humans in this region. The ability of the cestodes *G. osculata* to accumulate significantly higher PCB concentrations compared to fish organs was confirmed for the first time. This host-parasite model provides reliable information on the condition of the aquatic environment and can serve as a reference source for similar future comparative studies.

## Material and methods

### Study area and fish sampling

**Figure 5:**
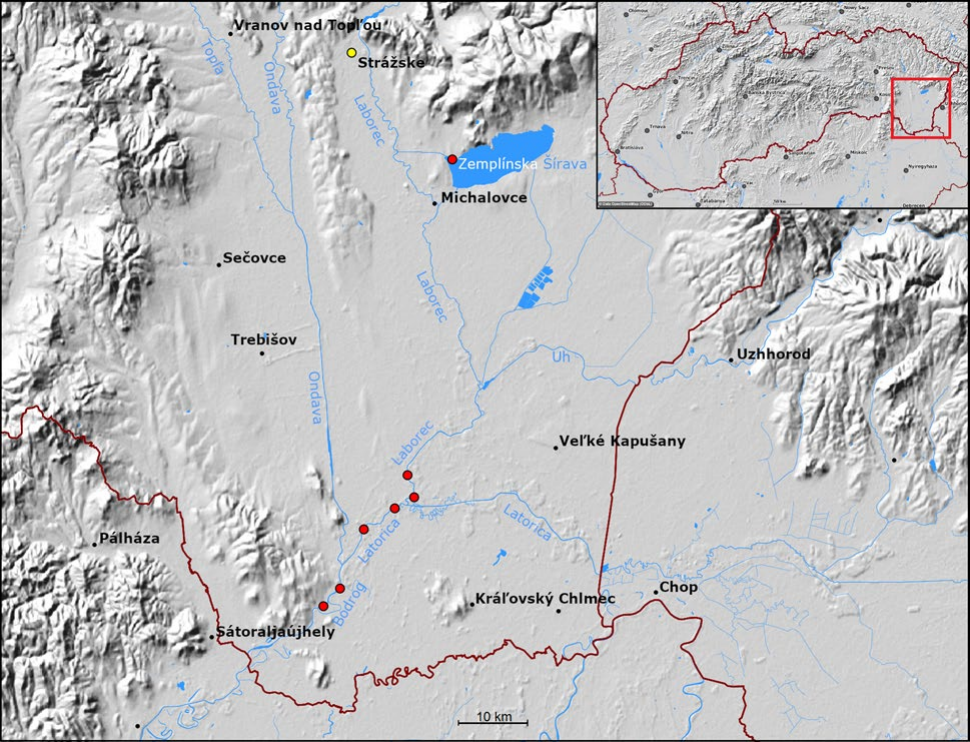
The map shows the sampling sites (red dots) of Wels catfish (*Silurus glanis*) from the Zemplínska Šírava reservoir and the Laborec, Latorica and Bodrog Rivers. The chemical factory in the town of Strážske in yellow. Details of the fish samples can also be found in Table S1 in the supplementary material.

The Bodrog River Basin area (Fig. 5) shared between Slovakia, Hungary and Ukraine, covers a total expanse of 11,522 km^2^, the Slovakian part of which occupies 7272 km^2^. The Bodrog River itself is 15.2 km long in this part of the basin, and towards the east it delimits the Slovak-Hungarian state border and flows into the Tisa River. The river arises from the confluence of the Ondava and Latorica Rivers, the main tributary of the latter being the Laborec River. The southern part of the Slovak area of the Bodrog River Basin lies at an elevation of 200–400 m above the sea level (m.a.s.l.) and has a predominantly flat character. A wetland of an international importance, the Latorica Protected Landscape Area (A Ramsar site), is located in the southern part of the Bodrog River Basin. It represents the large-scale lowland stretching along the Latorica River, the lower section of the Laborec and Ondava Rivers, and the upper part of the Bodrog River.

**Figure 6:**
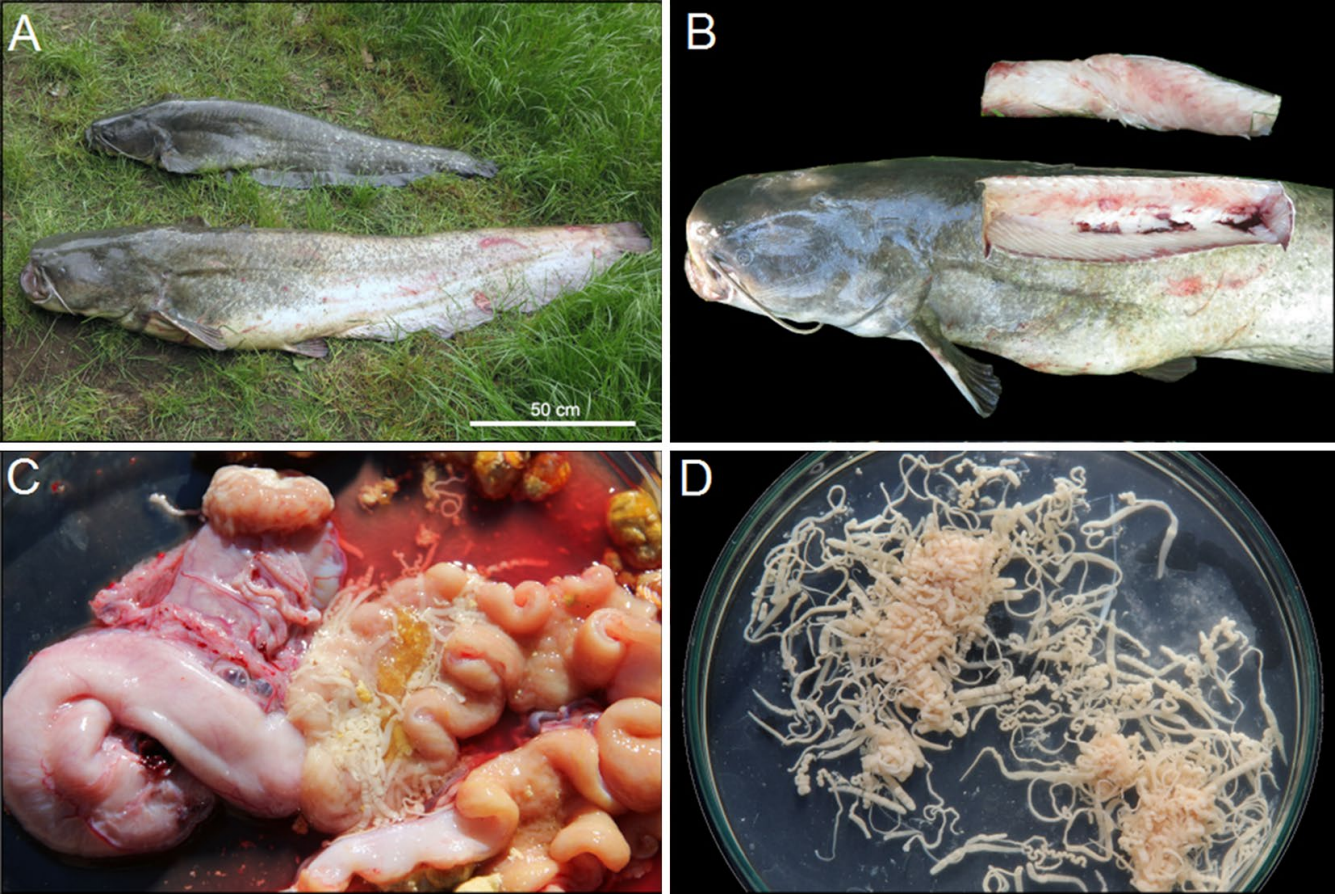
The Wels catfish (*Silurus glanis*) caught from the Zemplínska Šírava reservoir (**A**). Removal of part of the dorsal muscle for further determination of PCBs (**B**). The tapeworms (*Glanitaenia osculata*) in situ in the intestine of the catfish (**C**) and isolated tapeworms from the intestine washed in 0.9% saline solution (**D**).

The catfish (Wels catfish—*Silurus glanis* L.) were caught by electrofishing and fishing rod under permits issued by the Ministry of Environment of the Slovak Republic (No. 62/2020 and 30/2021) (Fig. 6). The animal study was reviewed and approved by the Ethics Committee of the Institute of Parasitology of the Slovak Academy of Sciences (Hlinkova 3, Košice, 04001, Slovakia), which also approved implementation of the project under approval No. 1/2020/PaU. All methods used in present study were carried out in accordance with relevant guidelines and regulations, and (Decree of the Ministry of the Slovak Republic no. 381/2018 Coll. and Act No. 216/2018 Coll. about fishing) we confirm that all methods are reported in accordance with ARRIVE guidelines^47^.

A total of 47 catfish were caught in May and June 2020 and 2021 at the following localities (Fig. 5): the water reservoir Zemplínska Šírava (48° 47′ 09.0″ N 21° 57′ 20.5″ E)—12 specimens; the Laborec River (48° 31′ 20.7″ N 21° 54′ 17.5″ E and 48° 30′ 38.9″ N 21° 54′ 33.2″ E)—11 specimens; the Latorica River (48° 29′ 55.8″ N 21° 52′ 46.8″ E and 48° 28′ 58.4″ N 21° 50′ 49.8″ E)—11 specimens; and the Bodrog River (48° 25′ 16.5″ N 21° 47′ 59.6″ E and 48° 24′ 07.6″ N 21° 46′ 38.2″ E)—13 specimens. The fish were transported to the laboratory, killed by severing the spinal cord, and the main biometric data (total length *L*_T_, cm; total weight *W*_T_, g) were measured. When possible, sex was identified by macroscopic observation of the gonads. The age of the fish was estimated from the growth curves of Alp et al.^48^. For confirmation, a vertebra was used to determine the age of some individuals.

The biological parameters (Supplementary Table S1 online) of the fish were as follows, mean ± SD (range): total length—81 ± 45.8 cm (38–193); weight—7708 ± 12,821 g (400–45,800); age—4.9 ± 4.7 years (in rivers and basin 1–17; in the Bodrog River 1–3). The muscles (the dorsal and abdominal parts separately, without skin and bones), intestine and the liver were removed with stainless steel instruments and kept frozen individually in plastic bags at -20 °C until further treatment. Parasites were isolated by an incomplete parasitological necropsy, focusing on the organs of the digestive system (Fig. 6). The cestodes were then isolated from the intestines of the fish, washed in 0.9% saline to remove remnants of intestinal content and counted. Specimens found in the same host individual were pooled and frozen separately at − 20 °C until further analysis. The epidemiological parameters (prevalence, intensity of infection; Supplementary Table S1 online) were determined following the concepts suggested by Bush et al.^49^.

### Analytical procedure

The quantification of non-dioxin like PCBs (NDL-PCBs) and their sum was performed according to Ahmed^50,51^. Six indicator PCB congeners (28, 52, 101, 138, 153 and 180) were analysed, and their cumulative analytical concentration was expressed as ∑PCBs. The PCBs were determined in the dorsal (n = 47) and abdominal muscle (n = 30) tissues, liver (n = 47) and intestine (n = 47) of the Wels catfish and in the cestodes (n = 12) using a gas chromatograph (GC) with an electron capture detector (Agilent 6890, Agilent Technologies, CA, USA). For chemical analysis, 2.0–37.5 g of fish matrices and 0.3–8.7 g of cestode tissue were used. The lipid content of all biological samples was determined as % lipid = 100 × (M_lipid_/M_sample_), where M_lipid_ is the weight of lipid extracted and M_sample_ is the weight of the analysed biological sample. The matrices were homogenised with anhydrous sodium sulphate and extracted with diethylether. The extracts were concentrated using a rotatory evaporator. The extractable lipid content was determined and purified on a H_2_SO_4_/silica column washed with a mixture of hexane and petrolether (94:6). The purified samples were evaporated, and the residue was dissolved in 0.5 ml of isooctane. Subsequently, 2 µl of the supernatant was injected and derivatised, using helium as the carrier gas in a 19091J-433W HP-5 capillary column (stationary phase: cross-linked 5% PH ME Siloxane, 30 m length × 0.25 mm diameter and 0.25 µm film thickness). The oven temperature of the GC was: initially 80 °C for 1 min, then increased to 180 °C at a rate of 30 °C min^−1^, 205 °C at 6 °C min^−1^, held for 4 min, and 290 °C at 20 °C min^−1^, held for 4.5 min.

The individual PCB congeners were identified on the basis of the retention times of a known standard and qualified by comparing the peak area with the corresponding peak in the standard mixture (PCB Mix 3 × 10 µg/mL in isooctane). The limits of detection (LOD), calculated as three times the signal-to-noise ratio, were about 0.3 ng g^−1^. The limits of quantification (LOQ) varied between 0.9 and 1.04 ng g^−1^. The recoveries were as follows: PCB 28—104.2%; PCB 52—105.3%; PCB 101—107.8%; PCB 138—108%; PCB 153—107.8% and PCB 180—108.7%. The PCB analyses were performed at the National Reference Laboratory, the State Veterinary and Food Institute in Košice, Slovakia. PCB concentrations in biological samples are expressed in ng g^−1^ wet weight (w.w.).

### Data analyses

PCB concentrations in fish muscles were compared with human consumption limits for freshwater fish meat^7^, and therefore they are expressed in ng g^−1^ w.w., and the influence of lipids was included as an explanatory variable in complex models. PCB concentrations in fish muscle, liver and intestine were partially correlated (Spearman correlation analysis) with the lipid content (p ˂ 0.05), but this trend was not proven in the parasites (p ˃ 0.05), and the intercept of the regression was not zero (see^52^).

Several PCB concentrations were below the detection limits (so-called censored data). If they were below 15%, the values were replaced by a number corresponding to half the detection limit before the statistical analysis^53^. Regarding the low number of samples and the fact that the data were censored, non-parametric statistical methods (Spearman correlation analysis, Mann–Whitney U-test) were applied using the Statistica software^54^, with statistical significance at p < 0.05.

The deviation of bioconcentration factors from the threshold value of 1 for each fish matrix (dorsal and abdominal muscle, liver and intestine) was tested using the non-parametric Wilcoxon matched pairs test. The Spearman’s correlation was used to find possible associations between pairs of variables (e.g. PCB concentrations and number of *G. osculata*; fish age/length/weight and number of *G. osculata;* lipid content and PCB concentration). The non-parametric Mann–Whitney U-test was used to determine whether there were significant differences between the sexes (males, females).

Four generalised linear mixed models (GLMMs) were used for testing the relationship between PCB concentrations (with a sufficient number of values above the LOD: ∑PCB, PCB 138, 153, 180) and variables of interest. Before analysis, the PCB values were log-transformed. The models were run in an R environment (v 4.1.0)^55^ with functions from the package 'brms'^56^. The following variables were used as fixed effects: parasitic infection (a two-level factor: infected, non-infected), age, lipid contents, a three-level factor (dorsal muscle, liver, intestine) for biological matrices and a four-level factor (the Zemplínska Šírava reservoir, Laborec, Latorica, Bodrog Rivers) for sampling sites. Abdominal muscle was not included in the complex models due to a small number of samples (n = 30). The identifiers of fish were added as random effects to avoid pseudoreplication. The models were fitted within a Bayesian framework using Markov chain Monte Carlo (MCMC) implemented in the function brm. MCMC was run using four chains of 30,000 iterations, each with 7500 iterations for each chain discarded as a burn-in for every model. The convergence diagnostics were explored via assessment of the Markov chain trace plots, autocorrelation plots and $\widehat{R}$ values. The non-informative priors were used for all fixed effects. The function *resp_cens* was used to encode models’ responses as censored data. The function *conditional_effects* was used to estimate the conditional effects of categorical factors.

## Supplementary Information

Supplementary Legends.

Supplementary Figure S1.

Supplementary Table S1.

## Data Availability

The original contributions presented in the study are included in the article/supplementary material; further inquiries can be directed to the corresponding authors.
