# Supplementary material for: A fish-parasite sentinel system in an assessment of the spatial distribution of polychlorinated biphenyls
Source: Sci Rep. 2023 Mar 30;13:5164. doi: 10.1038/s41598-023-31939-4 (PMC10063543; doi:10.1038/s41598-023-31939-4)
Supplement: Supplementary file 1 — Supplementary Legends. [file 41598_2023_31939_MOESM1_ESM.docx]

**Supplementary Figure S1 online**. Conditional effects of categorical predictors of four GLMM. Black dots indicate the size of the particular effect, and error bars show a 95% uncertainty interval of the effect.
