# Supplementary material for: A fish-parasite sentinel system in an assessment of the spatial distribution of polychlorinated biphenyls
Source: Sci Rep. 2023 Mar 30;13:5164. doi: 10.1038/s41598-023-31939-4 (PMC10063543; doi:10.1038/s41598-023-31939-4)
Supplement: Supplementary file 3 — Supplementary Table S1. [file 41598_2023_31939_MOESM3_ESM.docx]

**Table S1.** Biometric data of the Wels catfish (*Silurus glanis*) from the model localities and epidemiological parameters of the tapeworm *Glanitaenia osculata*. (*) means that the age of individual fish was confirmed with the using of vertebra.

| **Locality** | **Fish** | **Protocol No.** | **Length** | **Weight** | **Age** | **Gender** | **N of parasites** |
| --- | --- | --- | --- | --- | --- | --- | --- |
| Z. Šírava | *S. glanis* | 152/19 | 115 | 11,000 | 7 | M | 0 |
|  | (n=12) | 177/19 | 50 | 800 | 2* | M | 0 |
|  |  | 249/20 | 189 | 45,000 | 17* | M | 53 |
|  |  | 246/20 | 51 | 800 | 2 | F | 100 |
|  |  | 215/20 | 84 | 3,500 | 5 | F | 100 |
|  |  | 243/20 | 103 | 11,000 | 7* | M | 20 |
|  |  | 357/21 | 185 | 45,000 | 17 | F | 30 |
|  |  | 358/21 | 127 | 15,000 | 8 | M | 46 |
|  |  | 369/21 | 104 | 7,500 | 7 |  | 100 |
|  |  | 370/21 | 162 | 27,000 | 13* | F | 300 |
|  |  | 387/21 | 130 | 16,000 | 9 |  | 4 |
|  |  | 390/21 | 77 | 2,500 | 4* |  | 64 |
| Prevalence 83.3 % | Intensity of infection 81.7 (4–300) | | |  |  |  |  |
| Laborec | *S. glanis* | 297/20 | 193 | 45,800 | 17* | F | 0 |
|  | (n=11) | 298/20 | 127 | 12,300 | 9 | M | 1 |
|  |  | 299/20 | 68 | 2,000 | 4* | M | 0 |
|  |  | 300/20 | 65 | 1,500 | 3 | M | 2 |
|  |  | 401/21 | 110 | 9,000 | 8* | M | 0 |
|  |  | 402/21 | 61 | 1,630 | 3 | M | 0 |
|  |  | 403/21 | 47 | 700 | 2* | M | 0 |
|  |  | 404/21 | 44,5 | 530 | 1* | F | 0 |
|  |  | 405/21 | 42 | 430 | 1 |  | 0 |
|  |  | 407/21 | 130 | 10,000 | 9* |  | 0 |
|  |  | 410/21 | 73 | 2,500 | 4 | M | 0 |
| Prevalence 18.2 % | Intensity of infection 1.5 (1–2) | | |  |  |  |  |
| Latorica | *S. glanis* | 107/19 | 130 | 15,000 | 9 |  | 16 |
|  | (n=11) | 108/19 | 71 | 2,500 | 4* |  | 85 |
|  |  | 109/19 | 54 | 900 | 2 |  | 8 |
|  |  | 110/19 | 50 | 800 | 2 |  | 4 |
|  |  | 111/19 | 41 | 400 | 1 |  | 4 |
|  |  | 198/20 | 56 | 1,500 | 2 | M | 40 |
|  |  | 199/20 | 38 | 400 | 1* |  | 7 |
|  |  | 269/20 | 120 | 12,000 | 8 |  | 0 |
|  |  | 270/20 | 50 | 800 | 2 |  | 0 |
|  |  | 271/20 | 40 | 400 | 1 |  | 0 |
|  |  | 275/20 | 184 | 44,000 | 16* |  | 0 |
| Prevalence 63.6 % | Intesity of infection 23.4 (4–85) | | |  |  |  |  |
| Bodrog | *S. glanis* | 84/19 | 44 | 500 | 1 |  | 8 |
|  | (n=13) | 85/19 | 62 | 1,600 | 3* |  | 7 |
|  |  | 89/19 | 59 | 1,500 | 3 |  | 4 |
|  |  | 81/19 | 55 | 1,400 | 2 |  | 6 |
|  |  | 82/19 | 55 | 1,400 | 2* |  | 3 |
|  |  | 83/19 | 41 | 400 | 1 |  | 0 |
|  |  | 86/19 | 44 | 500 | 1* |  | 2 |
|  |  | 87/19 | 44 | 500 | 1 |  | 4 |
|  |  | 88/19 | 50 | 800 | 2* |  | 7 |
|  |  | 90/19 | 45 | 500 | 1 |  | 12 |
|  |  | 272/20 | 60 | 1,600 | 3* |  | 12 |
|  |  | 273/20 | 50 | 800 | 2* |  | 1 |
|  |  | 274/20 | 45 | 600 | 1 |  | 0 |
| Prevalence 84.6 % | Intensity of infection 6 (1–12) | | |  |  |  |  |
